# Relationship between Cardiovascular Calcium and Atrial Fibrillation

**DOI:** 10.3390/jcm11020371

**Published:** 2022-01-13

**Authors:** Sung Ho Lee, Mi Yeon Lee, Seung Yong Shin, Wang-Soo Lee, Sang-Wook Kim, Seung-Jung Park, June Soo Kim, Ki-Chul Sung

**Affiliations:** 1 Division of Cardiology, Department of Internal Medicine, Kangbuk Samsung Hospital, Sungkyunkwan University School of Medicine, Seoul 03181, Korea; 2 Division of Biostatistics, Department of R&D Management, Kangbuk Samsung Hospital, Sungkyunkwan University School of Medicine, Seoul 03181, Korea; my7713.lee@samsung.com; 3 Heart Research Institute, Cardiovascular-Arrhythmia Center, Chung-Ang University Hospital, Chung-Ang University, Seoul 06973, Korea; theshin04@cau.ac.kr (S.Y.S.); wslee1227@cau.ac.kr (W.-S.L.); swivus@gmail.com (S.-W.K.); 4 Division of Cardiology, Department of Medicine, Heart Vascular Stroke Institute, Samsung Medical Center, Sungkyunkwan University School of Medicine, Seoul 06351, Korea; orthovics@gmail.com (S.-J.P.); js58.kim@samsung.com (J.S.K.)

**Keywords:** coronary artery calcium score, aortic valve calcium, thoracic aorta calcium, atrial fibrillation

## Abstract

Coronary artery calcium score (CACS) is associated with increased risk of atrial fibrillation (AF). However, the relationship between the burden of CACS and extra-coronary calcium and the AF is unclear. This cross-sectional study retrospectively analyzed the data of 143,529 participants (74.9% men; mean age, 41.7 ± 8.6 years) who underwent health examination including non-contrast cardiac CT and electrocardiography, from 2010 to 2018 to evaluate the association between cardiac calcium and AF. AF was diagnosed in 679 participants. The prevalence of AF was significantly increased as the CACS increased (*p* < 0.01). Multivariable analysis adjusted for age, sex, body mass index, hypertension, diabetes, hyperlipidemia, smoking, alcohol, and history of coronary artery disease showed a significant association between a high CACS ≥1000 and AF (OR 2.26, 95% CI 1.07–4.77, *p* = 0.032). In a subgroup analysis of participants with a CACS ≥100, aortic valve and thoracic aorta calcium were significantly associated with AF (OR 3.49, 95% CI 1.57–7.77, *p* = 0.002 and OR 2.19, 95% CI 1.14–4.21, *p* = 0.01, respectively). High CACS was associated with AF, and extra-coronary atherosclerosis was associated with AF in participants with a moderate to very high CACS.

## 1. Introduction

Cardiovascular calcium is generally considered as a manifestation of atherosclerosis, and coronary artery calcium (CAC) is a useful marker for subclinical atherosclerosis of the coronary artery. Previous studies have shown that high CAC is strongly associated with the risk of future coronary artery disease (CAD) [1,2], cardiovascular events, mortality [3], and prediction of future atrial fibrillation (AF) [4,5,6,7]. Non-contrast cardiac computed tomography (CT) is a powerful noninvasive diagnostic imaging modality for the detection of CAC. Cardiac CT can be used to visualize the extent and location of coronary artery atherosclerosis as a function of the CAC burden [8,9,10].

AF is the most common clinical arrhythmia associated with stroke and heart failure. Traditional atherosclerosis risk factors, such as advanced age, male sex, hypertension, hyperlipidemia, diabetes mellitus, obesity, and smoking, also increase the risk of AF. Taken together, the close linkage of shared traditional atherosclerotic risk factors between CAC and AF suggests that CAC may also be associated with a high risk of AF [4,6]. Several studies have reported that a higher coronary artery calcium score (CACS) was associated with an increased risk of AF in both American and European populations [4,6]. However, the data supporting the association between CACS and AF in Asians are sparse. Furthermore, there are limited data supporting the link between extensive extra-coronary atherosclerosis and AF. We evaluated the relationship between cardiovascular calcium and AF to identify the effect of atherosclerosis on AF.

## 2. Materials and Methods

The study population underwent health examinations, including non-contrast cardiac CT and electrocardiography (ECG), between 2010 and 2018. Participants were excluded if they had a history of cancer (*n* = 4386) or previous cardiac surgery (*n* = 28) and missing data of body mass index (BMI) or cholesterol level (*n* = 8607). A total of 143,529 participants were eligible for this study. This study was approved by the Institutional Review Board of Kangbuk Samsung Hospital (IRB No. 2019-09-026). The requirement for informed consent was waived, as only anonymized retrospective data collected routinely during the health screening process were utilized.

**Figure 1:**
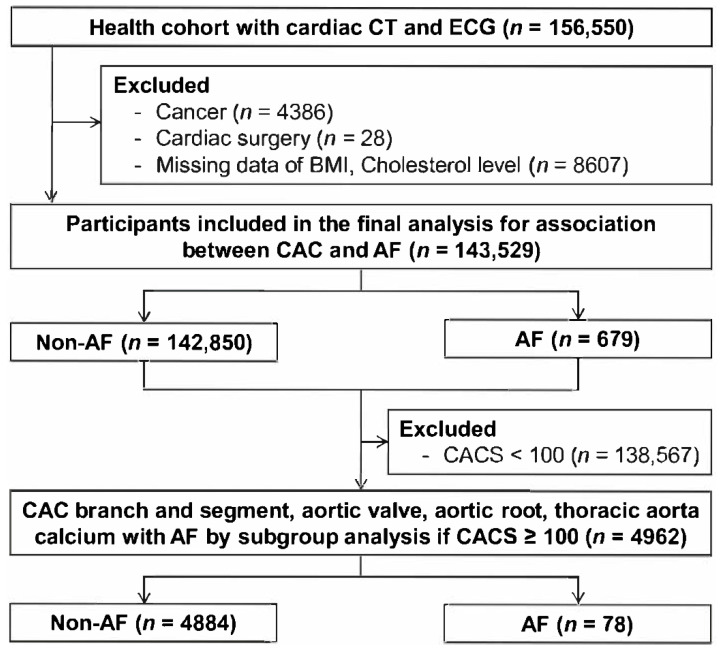
Flowchart of the study population. Abbreviations: ECG = electrocardiography; BMI = body mass index; AF = atrial fibrillation; CAC = coronary artery calcium; CACS = coronary artery calcium score.

We evaluated the association between CACS and AF in the 143,529 participants. We identified the coronary artery branches and segments carrying calcium, as well as the presence of aortic valve calcium (AVC), aortic root calcium (ARC), and thoracic aorta calcium (TAC) in 4962 participants with a CACS ≥100, since patients with a CACS ≥100 have a significantly lower survival rate [3,11] and high risk of stroke [12,13]. The flowchart of the study population is presented in Figure 1.

Data on medical history, current medications, and lifestyle factors were acquired using a structured self-administered questionnaire. Medical history included hypertension, diabetes mellitus, hyperlipidemia, chronic kidney disease (CKD), stroke, and CAD. AF was diagnosed using a 12-lead ECG on the day of non-contrast cardiac CT and medical checkup. The ECG was interpreted by an experienced cardiologist. A previous diagnosis of AF by a physician was also included. The CHA_2_DS_2_-VASc score assigns one point for a heart failure, hypertension, diabetes, vascular disease, age 65 to 74 years, and female gender, and two points for a history of stroke or peripheral embolism and age ≥75 years [14]. Lifestyle factors included smoking history (never, former, or current) and alcohol frequency (<3/week, ≥3/week).

Non-contrast cardiac CT scans were performed using a Lightspeed VCT XTe-64 slice MDCT scanner (GE Healthcare, Tokyo, Japan) using a standardized scanning protocol with a slice thickness of 2.5 mm, 400 ms rotation time, 120 kV tube voltage, and 124 mAS (310 mA × 0.4 s) tube current under ECG-gated dose modulation. The CAC Agatston scores were calculated by summing the CACS of all territories of the epicardial coronary artery: left main, left anterior descending (LAD), left circumflex (LCX), and right coronary artery (RCA) [15]. Cardiac CT was evaluated by an experienced radiologist. The interobserver reliability and intraobserver reliability for CACS were both excellent (intraclass correlation coefficient: 0.99) [16]. The original transaxial slices were inspected for the presence of significant calcium.

**Figure 2:**
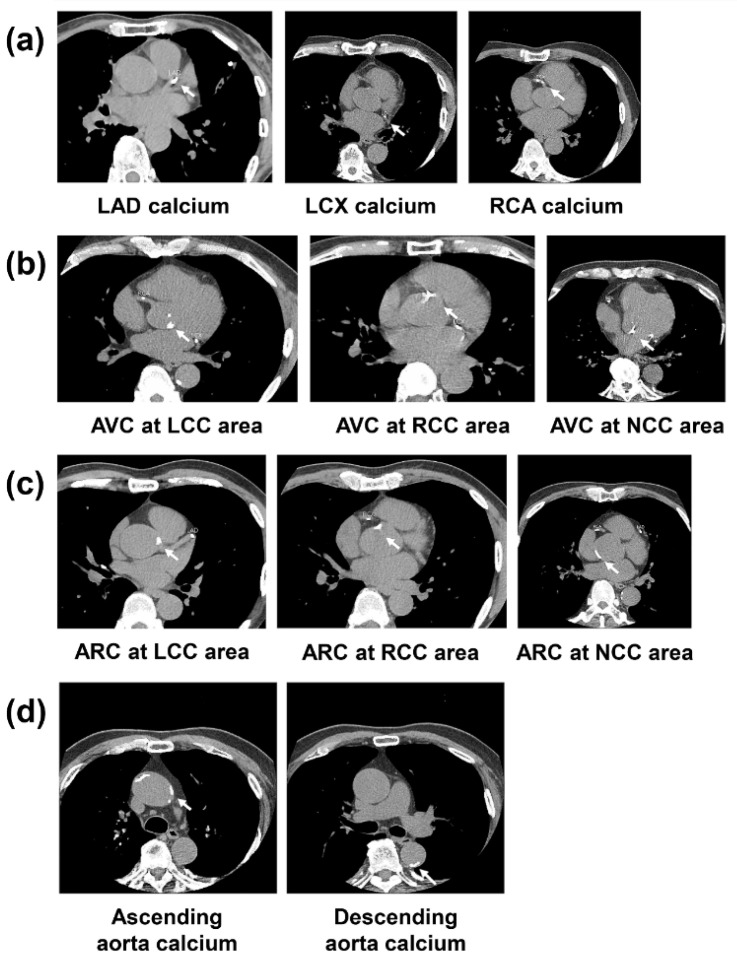
Cross-sectional cardiac CT of coronary artery calcium (**a**), aortic valve calcium (**b**), aortic root calcium (**c**), and thoracic aorta calcium (**d**). Abbreviations: ARC = aortic root calcium; AVC = aortic valve calcium; LAD = left anterior descending; LCC = left coronary cusp; LCX = left circumflex; NCC = noncoronary cusp; RCA = right coronary artery; RCC = right coronary cusp.

The CACS was categorized as follows: 0; mild, 1–99; moderate, 100–399; high, 400–999; very high, ≥1000 [17,18]. The coronary artery branch and the segment containing calcium in participants with a CACS ≥100 were evaluated according to the American Heart Association/American College of Cardiology guidelines [19]. The typical CAC in a coronary artery branch using cross-sectional cardiac CT is shown in Figure 2a.

AVC, ARC, and TAC can also be identified using non-contrast cardiac CT without any additional scanning or radiation. AVC was defined as calcium involving the aortic valve leaflets, points of attachment, and the aortic wall immediately connected to the calcified leaflets up to the level of the sinotubular junction (STJ). ARC was defined as calcium of the aortic root not immediately connected to the calcified leaflets, up to the level of the STJ. TAC was defined as any calcium in the ascending and descending aorta. The ascending thoracic aorta originates distal to the STJ, where the aortic root location is proximal to the STJ. The typical AVC, AVR, and TAC in cross-sectional cardiac CT is shown in Figure 2.

Continuous variables were expressed as the mean ± standard deviation, and categorical variables were expressed as percentages. Characteristics were compared across the five groups (0, 1–99, 100–399, 400–999, ≥1000) according to the CACS using analysis of variance (ANOVA) or χ^2^ tests. Post hoc comparisons of the groups based on CACS were performed with Scheffe’s multiple comparison test. Comparisons between two groups according to AF in participants with a CACS ≥100 were performed using either Student’s *t*-test or the χ^2^ test. Unadjusted logistic regression analysis was performed to evaluate the relationship between the CACS and AF using a CACS of 0 as the reference group. Multivariable models were constructed with incremental adjustments as follows: Model 1 was adjusted for age and sex; Model 2 was adjusted for Model 1 covariates plus BMI, hypertension, diabetes, smoking status, alcohol frequency, and hyperlipidemia; Model 3 included Model 2 and a previous history of CAD. The odds ratio (OR) and 95% confidence interval (CI) were estimated using a multivariable logistic regression with a bootstrap resampling method (1000 times). Receiver operating characteristic (ROC) curves were constructed for analysis of CACS to predict AF. Sensitivity, specificity, and positive and negative predictive value were measured. In subgroup analysis of subjects with a CACS ≥100, multivariable logistic regression was performed to evaluate the association of extra-coronary atherosclerosis as a function of parameters such as AVC, ARC, and TAC with AF. Statistical analysis of data was performed using PASW version 18 (SPSS, Chicago, IL, USA) and STATA version 17 (StataCorp LP, College Station, TX, USA). Statistical tests were two-tailed, and *p* < 0.05 was considered statistically significant.

## 3. Results

**Figure 3:**
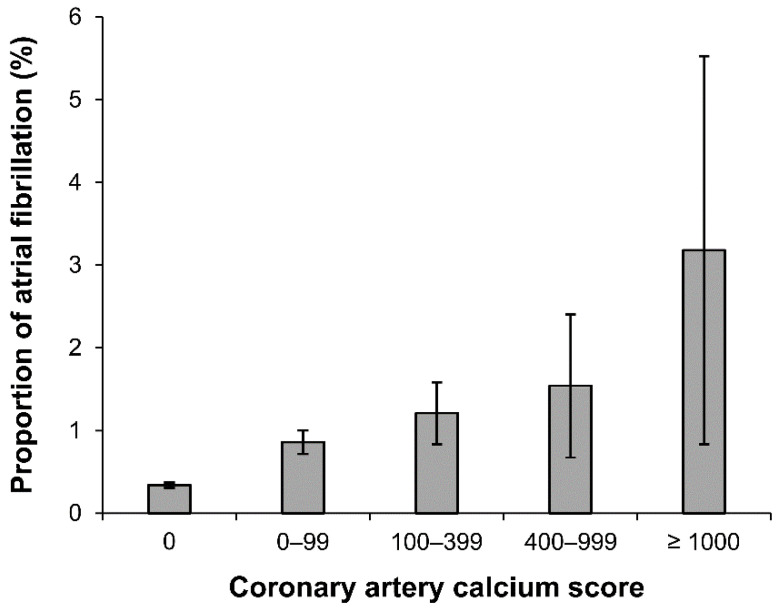
Proportion of atrial fibrillation based on coronary artery calcium score category. Error bar: 95% confidence interval.

Baseline clinical characteristics of the study population are shown in Table 1. The mean age of the participants was 41.7 ± 8.5 years, and 75% of participants were men. A total of 679 (0.47%) participants were diagnosed with AF. Of the 143,529 participants, 84.7% had a CACS of 0, 11.9% had a CACS of 1–99, 2.6% had a CACS of 100–399, 0.6% had a CACS of 400–999, and 0.2% had a CACS ≥1000. As expected, participants in the higher category of CAC were older, more often men, and had a higher CHA_2_DS_2_-VASc score, with a higher prevalence of hypertension, diabetes, hyperlipidemia, CKD, previous history of stroke, and CAD. The prevalence of AF increased in the higher CACS categories (Figure 3).

Table 2 shows the association between CAC categories and AF. According to an unadjusted analysis, the OR of AF increased from 2.60 (95% confidence interval (CI) 2.16–3.11, *p* < 0.001) among participants with a CACS of 1–99 to 10.24 (95% CI 5.57–18.83, *p* < 0.001) in participants with a CACS of ≥1000 compared with those without CAC. In the fully adjusted Model 3, including BMI, hypertension, diabetes, smoking status, alcohol frequency, hyperlipidemia, and previous history of CAD, only a very high CACS (≥1000) was independently associated with AF (OR 2.26, 95% CI 1.07–4.77, *p* = 0.032).

**Figure 4:**
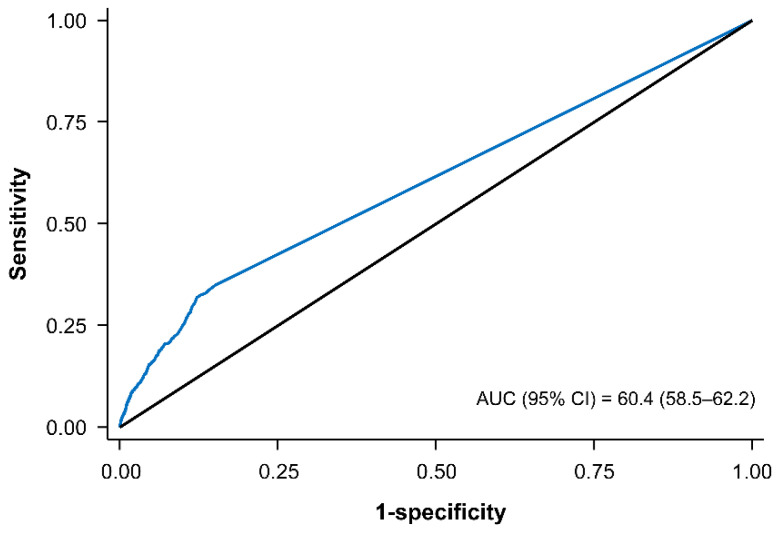
Receiver operating characteristic curve for coronary artery calcium score to predict atrial fibrillation.

The ROC curves of CACS for AF showed that area under the curve was 0.60 (95% CI: 0.58–0.62) as a continuous variable of CACS >0 (Figure 4). The predictive value of CACS for AF according to different categorical values is shown in the Table 3.

The coronary artery branch and the segment calcium according to AF in participants with moderate to very high CACS ≥100 (*n* = 4962) are shown in Table 4. The CACS of the RCA was significantly higher in participants with AF compared with those without AF (244.3 ± 415.6 vs. 134.1 ± 242.9, *p* = 0.02). Proximal RCA calcium (46.2% vs. 26.2%, *p* = 0.001) was more frequently observed in AF participants than in those without AF.

In 4962 participants with CACS ≥100, AVC (24.4% vs. 7.6%, *p* < 0.001), ARC (24.4% vs. 11.7%, *p* = 0.002), and TAC (60.3% vs. 32.5%, *p* < 0.001) were more frequently found in the AF group than in those without AF (Table 3). Multivariable analysis showed that AVC (OR 3.49, 95% CI 1.57–7.77, *p* = 0.002) and TAC (OR 2.19, 95% CI 1.14–4.21, *p* = 0.01) were significantly associated with AF (Table 5).

## 4. Discussion

The main findings of this study were as follows: (1) severe atherosclerotic burden, represented as high CACS (≥1000), was significantly associated with AF; (2) in particular, the CACS of the proximal RCA was higher in AF; (3) AVC and TAC were incrementally associated with AF in the moderate to very high CACS category (CACS ≥100).

Previous studies showed an association between high CACS and AF in American [6] and European data [4], but the Asian data about these relationships are scarce. Our study consistently showed a significant association between heavy CAC and AF in a large Asian population of middle-aged individuals. After adjusting for traditional risk factors, severe atherosclerotic burden of coronary artery (CACS ≥1000) was independently associated with AF. Although we adjusted for the previous history of CAD, similar results showed that a higher CACS (≥1000) was independently associated with AF, which suggested an association between subclinical heavy coronary atherosclerosis and AF even without significant luminal stenosis.

CAC impairs endothelial function and causes diastolic dysfunction without significant luminal stenosis before clinically manifesting as CAD [20]. These microvascular and diastolic dysfunction with increased ventricular filling pressure could increase atrial remodeling and stretching, which may result in further electrical inhomogeneity of the atrial conduction and refractoriness, resulting in an enlarged left atrium (LA) [21,22]. Previous studies reported that coronary branches arising from both the initial segment of the RCA and the LCX perfuse blood to the posterior LA, which are crucial areas for the trigger and maintenance of AF in isolated sheep heart [23,24]. Our study showed a higher CACS of proximal RCA in the AF group; however, whether this CACS of RCA influenced LA remodeling is uncertain.

The cardiovascular death outcome and all-cause mortality were significantly increased by high CACS grades (CACS ≥100) [3,11]. In our study, the extensive extra-coronary atherosclerosis including AVC and TAC in the participants with moderate to very high CACS (CACS ≥100) was significantly associated with AF after adjusting for traditional atherosclerosis risk factors. The mechanism underlying the association between extra-coronary calcium and AF is not clear. However, increased vascular stiffness related to mechanical or shear stress from calcified aortic valve and aorta might contribute to left-atrial enlargement. Further studies are needed to determine the effect of vascular stiffness on the LA as a function of LA pressure or degree of aortic stenosis. The clinical implication of our study is that screening for AF might be effective in participants with very high CACS or combined extra-coronary atherosclerosis, including AVC or TAC. As the risk of AF could increase in participants with high CACS, there could be a possibility of early detection of subclinical AF if frequent and prolonged monitoring of ECG is performed in these participants. These high-CACS participants tend to have multiple morbidities, which increase the stroke risk [12,13]. Optimal treatment such as anticoagulation could be considered if AF is early diagnosed in participants with high CACS and AF.

This study had several limitations. Firstly, we could not establish a causal relationship between CACS and AF because of the cross-sectional nature of the study. Secondly, our study population consisted of middle-aged participants in a health checkup program, which limits the generalizability to the entire community-based population. Thirdly, paroxysmal AF may have been missed by a single episode of 12-lead ECG. A single ECG for detection of paroxysmal AF could underestimate the relationship between CAC and AF. Furthermore, we were unable to acquire echocardiographic data suggesting structural remodeling, such as LA enlargement or diastolic dysfunction and severity of aortic stenosis. Fourthly, we could not assess the severity of calcium in the aortic vascular structure, such as the aortic valve, aortic root, and thoracic aorta calcium score. Fifthly, we could not estimate the effects of medications, such as statins or warfarin, that could affect vascular calcium. Lastly, the data of CACS exhibited a skewed distribution; thus, the possibility of unmeasured or residual confounding factors cannot be excluded.

In conclusion, our study found that severe CAC was significantly associated with AF, and extra-coronary atherosclerosis was additionally associated with AF in moderate to very high CACS. Opportunistic screening for AF might be reasonable in participants with advanced atherosclerosis. Further longitudinal studies are needed to validate these relationships.

## Figures and Tables

**Table 1:** Baseline clinical characteristics according to coronary artery calcium score.

Characteristics	Coronary Artery Calcium Score (*n* = 143,529)						
	All (*n* = 143,529)	0 (*n* = 121,512)	1–99 (*n* = 17,055)	100–399 (*n* = 3730)	400–999 (*n* = 926)	≥1000 (*n* = 306)	*p*-Value
Age, years	41.77 ± 8.56	40.5 ± 7.7	48.8 ± 8.9	53.9 ± 9.4	57.7 ± 9.6	59.6 ± 10.5	<0.001
Sex (male)	107,541 (74.9)	88,181 (72.5)	14,982 (87.8)	3285 (88.1)	811 (87.6)	282 (92.2)	<0.001
Body mass index, kg/m^2^	24.44 ± 3.33	24.2 ± 3.3	25.2 ± 3.1	25.4 ± 3.1	25.2 ± 3.1	25.3 ± 3.2	<0.001
Hypertension	25,242 (17.6)	16,485 (13.6)	6034 (35.4)	1906 (51.1)	603 (65.1)	214 (70.4)	<0.001
Diabetes mellitus	9370 (6.5)	5452 (4.5)	2521 (14.8)	946 (25.4)	310 (33.5)	141 (46.2)	<0.001
Hyperlipidemia	30,431 (23.4)	21,993 (20.0)	6089 (39.1)	1721 (50.0)	483 (56.8)	145 (53.5)	<0.001
Heart failure	296 (0.2)	209 (0.2)	56 (0.3)	16 (0.4)	5 (0.5)	10 (3.3)	<0.001
Chronic kidney disease	3367 (2.3)	2790 (2.3)	432 (2.5)	103 (2.8)	28 (3.0)	14 (4.6)	0.004
Coronary artery disease	1259 (0.9)	691 (0.6)	297 (1.7)	158 (4.2)	62 (6.7)	51 (16.8)	<0.001
Stroke	797 (0.6)	462 (0.4)	190 (1.1)	88 (2.4)	33 (3.6)	24 (7.9)	<0.001
AF	679 (0.4)	441 (0.4)	160 (0.9)	51 (1.4)	16 (1.7)	11 (3.6)	<0.001
CHA_2_DS_2_-VASc	0.5 ± 0.6	0.4 ± 0.6	0.7 ± 0.8	1.0 ± 1.0	1.4 ± 1.1	1.8 ± 1.2	<0.001
Total cholesterol, mg/dL	196.93 ± 34.88	196.4 ± 33.9	202.0 ± 38.3	192.9 ± 41.8	187.7 ± 41.5	176.3 ± 43.1	<0.001
Triglycerides, mg/dL	133.42 ± 91.63	129.8 ± 89.3	153.5 ± 98.8	153.4 ± 113.1	150.6 ± 94.0	132.7 ± 75.4	<0.001
LDL-C, mg/dL	128.99 ± 32.77	128.2 ± 31.9	135.7 ± 35.4	127.0 ± 38.5	121.1 ± 38.0	112.4 ± 40.2	<0.001
HDL-C, mg/dL	55.77 ± 14.86	56.3 ± 14.9	52.5 ± 13.5	52.6 ± 13.9	53.1 ± 14.5	52.3 ± 14.5	<0.001
Glucose, mg/dL	98.16 ± 16.65	96.8 ± 14.6	103.9 ± 21.9	109.4 ± 28.1	112.1 ± 28.5	115.5 ± 33.7	<0.001
HbA1c	5.6 ± 0.5	5.5 ± 0.5	5.8 ± 0.7	6.0 ± 0.9	6.1 ± 1.0	6.4 ± 1.2	<0.001
hs-CRP, mg/L	0.11 ± 0.34	0.11 ± 0.34	0.11 ± 0.32	0.13 ± 0.36	0.13 ± 0.42	0.15 ± 0.52	<0.001
Alcohol frequency							<0.001
None	11,558 (8.5)	9668 (8.3)	1398 (8.7)	361 (10.4)	93 (11.0)	38 (14.2)	
<3/week	100,640 (73.6)	86,771 (81.5)	11,047 (74.9)	2211 (71.2)	522 (69.2)	149 (64.8)	
≥3/week	24,577 (18.0)	19,665 (18.5)	3705 (25.1)	894 (28.8)	232 (30.8)	81 (35.2)	
Self-reported smoking status							<0.001
Never	60,900 (42.4)	54,981 (46.8)	4748 (29.0)	913 (26.0)	199 (23.4)	59 (21.9)	
Former smoker	48,307 (33.6)	38,977 (33.2)	7143 (43.6)	1622 (46.1)	421 (49.5)	144 (53.3)	
Current smoker	29,298 (20.4)	23,530 (20.0)	4490 (27.4)	981 (27.9)	230 (27.1)	67 (24.8)	

Continuous variables are presented as the mean ± standard deviation and categorical variables are presented as numbers (percentage). CHA_2_DS_2_-VASc indicates heart failure, hypertension, age ≥75 (doubled), diabetes mellitus, prior stroke or peripheral embolism (doubled), vascular disease, age 65 to 74, female. Abbreviations: AF = atrial fibrillation; LDL-C = low-density lipoprotein cholesterol; HDL-C = high-density lipoprotein cholesterol; HbA1c = glycated hemoglobin; hs-CRP = high sensitivity C-reactive protein.

**Table 2:** Multivariable logistic regression analyses for the relationship of coronary artery calcium score and atrial fibrillation in total 143,529 participants.

CACS	Univariable		Multivariable					
			Model 1		Model 2		Model 3	
	OR (95% CI)	*p*-Value	OR (95% CI)	*p*-Value	OR (95% CI)	*p*-Value	OR (95% CI)	*p*-Value
0	1 (reference)		1 (reference)		1 (reference)		1 (reference)	
1–99	2.60 (2.16–3.11)	<0.001	1.34 (1.09–1.64)	0.004	1.27 (0.99–1.68)	0.050	1.29 (0.98–1.56)	0.052
100–399	3.80 (2.84–5.09)	<0.001	1.36 (0.98–1.87)	0.062	1.30 (0.95–1.78)	0.106	1.29 (0.89–1.88)	0.183
400–999	4.83 (2.92–7.98)	<0.001	1.33 (0.76–2.35)	0.323	1.27 (0.76–2.14)	0.360	1.26 (0.66–2.41)	0.478
≥1000	10.24 (5.57–18.83)	<0.001	2.36 (1.17–4.87)	0.017	2.29 (1.28–4.11)	0.005	2.26 (1.07–4.77)	0.032

Model 1: adjusted for age, sex. Model 2: Adjusted as in Model 1 and for body mass index, hypertension, diabetes, smoking status, alcohol frequency, and hyperlipidemia. Model 3: adjusted as in Model 2 and for history of coronary artery disease. Abbreviations: CACS = coronary artery calcium score; CI = confidence interval; OR = odds ratio.

**Table 3:** Predictive accuracy of CACS for AF risk.

CACS	Sensitivity (%)	Specificity (%)	Positive Predictive Value (%)	Negative Predictive Value (%)
1–99	26.6	87.8	0.9	99.6
100–399	10.4	97.1	1.4	99.6
400–999	3.5	99.3	1.7	99.6
≥1000	2.4	99.8	3.6	99.6

Abbreviations: CACS = coronary artery calcium score, AF = atrial fibrillation.

**Table 4:** Cardiovascular calcium distribution according to atrial fibrillation (CACS ≥100).

Cardiovascular Calcium	All (*n* = 4962)	Non-AF (*n* = 4884)	AF (*n* = 78)	*p*-Value
CACS	366.83 ± 456.20	364.02 ± 451.93	542.61 ± 651.80	0.01
LAD calcium score	187.88 ± 239.08	188.24 ± 239.33	225.55 ± 261.01	0.17
Proximal	3487 (70.3)	3434 (70.3)	53 (67.9)	0.96
Diagonal	1561 (31.5)	1541 (31.6)	20 (25.6)	0.51
Mid	3318 (66.9)	3278 (67.1)	40 (51.3)	0.01
Distal	343 (6.9)	343 (7.0)	0	0.05
LCX calcium score	55.72 ± 111.78	55.84 ± 112.15	66.55 ± 110.43	0.40
Proximal	1415 (28.5)	1395 (28.6)	20 (25.6)	0.61
OM	970 (19.5)	961 (19.7)	9 (11.5)	0.19
Distal	912 (18.4)	894 (18.3)	18 (23.1)	0.28
RCA calcium score	135.23 ± 246.60	134.12 ± 242.98	244.31 ± 415.67	0.02
Proximal	1316 (26.5)	1280 (26.2)	36 (46.2)	0.001
Mid	2647 (53.3)	2607 (53.4)	40 (51.3)	0.90
Distal	1397 (28.2)	1374 (28.1)	23 (29.5)	0.98
Aortic valve calcium	388 (7.8)	369 (7.6)	19 (24.4)	<0.001
Aortic root calcium	592 (11.9)	573 (11.7)	19 (24.4)	0.002
Thoracic aorta calcium	1634 (32.9)	1587 (32.5)	47 (60.3)	<0.001

Continuous variables are presented as the mean ± standard deviation and categorical variables are presented as numbers (percentage). Abbreviations: AF = atrial fibrillation; CACS = coronary artery calcium score; LAD = left anterior descending; LCX = left circumflex; OM = obtuse marginal; RCA = right coronary artery.

**Table 5:** Multivariable logistic regression analyses for the relationship of aortic valve, aortic root, thoracic aorta calcium, and atrial fibrillation in 4962 participants (CACS ≥100).

	Univariable		Multivariable					
			Model 1		Model 2		Model 3	
	OR (95% CI)	*p*-Value	OR (95% CI)	*p*-Value	OR (95% CI)	*p*-Value	OR (95% CI)	*p*-Value
TAC	2.60 (1.60–4.21)	<0.001	2.21 (1.31–3.74)	0.003	2.19 (1.14–4.21)	0.018	2.19 (1.14–4.21)	0.010
AVC	2.76 (1.47–5.15)	0.001	2.42 (1.28–4.57)	0.006	3.46 (1.55–7.71)	0.002	3.49 (1.57–7.77)	0.002
ARC	1.11 (0.59–2.08)	0.730	1.02 (0.54–1.92)	0.920	0.88 (0.38–2.03)	0.760	0.88 (0.38–2.03)	0.760

Model 1: adjusted for age, sex. Model 2: adjusted as in Model 1 and for body mass index, hypertension, diabetes, smoking status, alcohol frequency, and hyperlipidemia. Model 3: adjusted as in Model 2 and for history of coronary artery disease. Abbreviations: ARC = aortic root calcium; AVC = aortic valve calcium; CI = confidence interval; OR = odds ratio; TAC = thoracic aorta calcium.

## Data Availability

The datasets generated during and/or analyzed during the current study are not publicly available due to maintaining patient confidentiality but are available from the corresponding author on reasonable request.
